# Psychological health problems among Syrians during war and the COVID-19 pandemic: national survey

**DOI:** 10.1192/bji.2021.16

**Published:** 2021-08

**Authors:** Fatema Mohsen, Batoul Bakkar, Sara Melhem, Samar Aldakkak, Dana Mchantaf, Marah Marrawi, Youssef Latifeh

**Affiliations:** 1 Faculty of Medicine, Syrian Private University, Damascus, Syria. Email: fatemamohsena@gmail.com; 2 Faculty of Medicine, Syrian Private University, Damascus, Syria; 3 Faculty of Medicine, Syrian Private University, Damascus, Syria; 4 Faculty of Medicine, Syrian Private University, Damascus, Syria; 5 Faculty of Medicine, Syrian Private University, Damascus, Syria; 6 Department of Statistics, Syrian Private University, Damascus, Syria; 7 Department of Psychiatry, Faculty of Medicine, Syrian Private University, Damascus, Syria; and Department of Psychiatry, Faculty of Medicine, Damascus University, Damascus, Syria

**Keywords:** Anxiety disorders, depressive disorders, low and middle income countries, national survey, war and pandemic

## Abstract

This study aims to assess the prevalence of depression and anxiety during the COVID-19 outbreak embedded within the war in Syria. A web-based cross-sectional survey design was employed. The 9-item Patient Health Questionnaire and the 7-item Generalized Anxiety Disorder scale revealed a high prevalence of depressive (*n* = 3326; 83.4%) and anxiety symptoms (*n* = 2777; 69.6%) among the 3989 participants. Multivariable logistic regression analysis was performed to identify factors associated with depression and anxiety. There is an urgent need within the healthcare system in Syria to provide mental healthcare to alleviate acute mental health disturbances and associated physical health perceptions among Syrians.

The Syrian conflict, now spanning a decade, paralleled with the domestically spreading coronavirus disease 2019 (COVID-19), is threatening the lives of civilians across Syria. The mental health of Syrians has been immensely challenged during the viral pandemic that emerged in this ongoing crisis. Healthcare systems have been pushed to breaking point to deal with the pandemic, with the ever-increasing number of COVID-19-related deaths and confirmed cases across the country leading to public trepidation. This sudden outbreak of a deadly virus is bound to trigger anxiety, depression and other mental health issues among the population. Lack of effective treatment, fears of death and contagion, losing loved ones, social isolation, media misinformation overload, depletion of stocks of personal protection equipment, panic buying and inadequate psychosocial support may all adversely affect the individual's mental health. Worldwide, levels of anxiety symptoms have ranged from 6.3 to 50.9% and depressive symptoms from 17.2 to 53.5% during the COVID-19 pandemic.^1^

The prolonged conflict in Syria has exposed civilians to grave human rights violations, including killing, maiming, sexual assault, torture, and displacement, where 89% of the population are living in extreme poverty.^2^ Infrastructures, including hospitals, schools and public services, are being systematically decimated. For instance, the Syrian Ministry of Health provides treatment for mental illness and substance misuse in three hospitals: Ibn Sina Hospital (rural Damascus), Ibn Rushd Hospital (Damascus) and Ibn Khaldoun Hospital (Aleppo); the last was bombarded on 25 December 2012.^3^ One million Syrians are estimated to suffer from severe psychiatric disorders, with only 80 psychiatrists working in Syrian territories (1 per 100 000 population) in 2018.^2,3^ Assuming that psychiatrists work 5 days/week for 52 weeks/year, and that each doctor can follow up on 15 patients/day and that they do not follow up on each patient >3 times/year, the total number of patients that the 80 psychiatrists can follow up on annually is 104 000; that leaves 90% of patients unattended, untreated and unmanaged. The combination of the above is set to overwhelm health services.^3^

Currently, there are no published studies on the simultaneous impact of war and pandemics; however, a recent systematic review on the mental health of Syrian refugees and Syrians revealed that depression ranged from 11 to 49% and anxiety from 49 to 55%.^4^ A recently published study showed alarming numbers, where 60% of the Syrian population are suffering from symptoms consistent with moderate to severe mental disorder.^5^ We describe here the first study that aimed to provide an estimated prevalence of depressive and anxiety symptoms among Syrians during the civil war and COVID-19 pandemic. The objective was to identify potential factors associated with depressive and anxiety symptoms.

## Method

This web-based cross-sectional study was conducted in May 2020. All participants aged 18 and above, residing in Syria, who completed the survey were included in the study. The questionnaire was distributed through various social media platforms. The Arabic versions of the 9-item Patient Health Questionnaire (PHQ-9) and the 7-item Generalized Anxiety Disorder (GAD-7) scale were used to evaluate the symptoms of depression and anxiety respectively.^6^ The PHQ-9 and GAD-7 cut-off scores were both 0–4.^7,8^ The study was ethically approved by the Institutional Review Board of the Faculty of Medicine, Syrian Private University. The survey is shown in supplementary Appendix 1, available at https://dx.doi.org/10.1192/bji.2021.16. All participants provided written informed consent to participate in the study. Data analysis was conducted using the Statistical Package for Social Sciences version 25.0 for Windows. The chi-squared test was applied to describe the relationship between sociodemographic variables and the severity of anxiety and depression. Binary logistic regression analysis was conducted to determine the significant predictors of depressive and anxiety symptoms using the sociodemographic variables as the independent variables.

## Results

Of the 5000 total participants invited to take part in the study, the final sample was 3989 participants (a response rate of 79.8%). Sociodemographic characteristics are displayed in Table 1.

**Table 1:** Sociodemographic characteristics: (*n* = 3989)

	*n* (%)
Gender	
Male	1054 (26.4)
Female	2935 (73.5)
Age, years	
18–25	2870 (71.9)
26–34	685 (17.2)
35–44	261 (6.5)
45–54	121 (3)
>55	52 (1.4)
Social status	
Single	3096 (77.6)
Married	714 (17.9)
Other	179 (4.5)
Economic status	
Excellent	251 (6.3)
Good	1800 (45.1)
Moderate	1522 (38.1)
Poor	416 (10.4)
Chronic disease(s)	
Yes	556 (13.9)
No	3433 (86)
Education	
Primary school	21 (0.5)
Intermediate school	115 (2.9)
Secondary school	370 (9.3)
College/university	3271 (82)
Master's degree	185 (4.6)
PhD	27 (0.7)
Occupation	
Healthcare worker	259 (6.5)
Government institution	239 (6)
Private institution	202 (5.1)
Business	202 (5.1)
Military	35 (0.9)
Student	2397 (60.1)
Other	655 (16.4)
Household members	
Alone	54 (1.4)
1–5	2474 (62)
>5	1461 (36.6)

The prevalence of depressive and anxiety symptoms among participants was 3326 (83.4%) and 2777 (69.6%) respectively. Depression severity: none, 663 (16.6%); mild, 1150 (28.8%); moderate, 1006 (25.2%); moderately severe, 684 (17.2%); and severe, 486 (12.2%). Anxiety severity: none, 1212 (30.4%); mild, 1293 (32.4%); moderate, 805 (20.2%); and severe 679 (17.0%).

Depression scores were significantly higher among participants who were single (*P* < 0.0001), had college/university level education (*P* < 0.0001), were students by occupation (*P* < 0.0001), lived with 1–5 household members (*P* < 0.0001), had a history of chronic disease(s) (*P* < 0.0001) and were of good economic status (*P* < 0.0001). Anxiety scores were significantly higher among those with college/university level education (*P* < 0.0001), were students by occupation (*P* < 0.0001), lived with 1–5 household members (*P* = 0.014), had a history of chronic disease(s) (*P* < 0.0001) and were of good economic status (*P* < 0.0001) (supplementary Appendix 2).

Multiple logistic regression analysis showed that the following factors were significantly associated with depressive and anxiety symptoms: female gender; age 18–25 years; occupation in private, business and other sectors, as well as students; poor, moderate and good economic status; and having chronic disease(s), (Table 2).

**Table 2:** Multiple logistic regression analysis of depressive and anxiety symptoms

		*P*	OR	95% CI for OR	
				Lower	Upper
**Depression**					
Gender	Female *v.* male	<0.0001	1.653	1.355	2.017
Age	26–34 *v.* 18–25	0.015	0.713	0.544	0.936
	35–44 *v.* 18–25	<0.0001	0.430	0.297	0.622
	45–54 *v.* 18–25	<0.0001	0.298	0.184	0.482
	55 and above *v.* 18–25	<0.0001	0.156	0.080	0.308
Occupation	Private *v.* healthcare worker	0.010	1.825	1.152	2.891
	Business *v.* healthcare worker	0.003	2.058	1.280	3.310
	Student *v.* healthcare worker	<0.0001	2.315	1.658	3.234
	Other *v.* healthcare worker	0.001	1.809	1.259	2.599
Economic status	Poor *v.* excellent	<0.0001	4.771	2.956	7.698
	Moderate *v.* excellent	<0.0001	2.075	1.473	2.922
	Good *v.* excellent	0.011	1.531	1.101	2.130
Chronic disease(s)	Yes *v.* no	<0.0001	2.990	2.120	4.215
**Anxiety**					
Gender	Female *v.* male	<0.0001	1.476	1.263	1.725
Age	26–34 *v.* 18–25	0.001	0.708	0.577	0.869
	35–44 *v.* 18–25	<0.0001	0.328	0.234	0.459
	45–54 *v.* 18–25	<0.0001	0.243	0.154	0.384
	55 and above *v.* 18–25	<0.0001	0.189	0.100	0.357
Education	University/college, Master's, PHD *v.* primary, intermediate, secondary	<0.0001	0.624	0.488	0.798
Occupation	Healthcare worker *v.* government, private, business, military, student, other	0.021	0.725	0.552	0.953
Economic status	Poor *v.* excellent	<0.0001	6.339	4.390	9.153
	Moderate *v.* excellent	<0.0001	3.140	2.371	4.160
	Good *v.* excellent	0.008	1.451	1.101	1.911
Chronic disease(s)	Yes *v.* no	<0.0001	2.492	1.941	3.198

## Discussion

The prevalence of depressive 3326 (83.4%) and anxiety 2777 (69.6%) symptoms among Syrians was significantly higher compared with the prevalence of anxiety in 17 studies (31.9%) and the prevalence of depression in 14 studies (33.7%).^1^ The high prevalence of depressive and anxiety symptoms found in our study surpasses World Health Organization prevalence estimations (of 3.9% and 4.3% respectively).^9^ A potential explanation for this disparity is that exposure to multiple stressors, including war and pandemic, may have triggered mental disorders among Syrians.

Literature has revealed that Syria's lack of mental healthcare services provided by psychologists and psychiatrists results in limited access to mental healthcare for many distressed individuals. Scarcity of online and social media support in the form of publications, resources, posts, guidelines, videos and online group chats is likely to exacerbate the problem.^2,3^ Such support can alleviate mental health problems caused by risk factors such as exposure to COVID-19.

All participants presenting with mild (1150; 28.8%) and moderate (1006; 25.2%) depression and mild (1293; 32.4%) and moderate (805; 20.2%) anxiety require clinical assessment for symptom duration and functional disability to determine the necessity of treatment. As over half the population (54% for depression and 52.6% for anxiety) requires a scheduled clinical assessment, with a total population of 18 284 423, this means that over 9 million people require mental health services. The lack of psychiatrists, hospitals, medical capacity and clinics projects ominous outcomes for Syrians’ mental health.

Participants with moderately severe (684; 17.2%) and severe (486; 12.2%) depression and severe (679; 17.0%) anxiety may require further assessment and active treatment with psychotherapy and/or medications. In 2005, the health expenditure per capita in Syria was US$58, which is extremely low in comparison with the UK (US$2900).^10^ As the country is drowning in debts from the effects of the rampant war, the health expenditure per capita is likely to be significantly lower in the coming years. In 2013, the World Federation for Mental Health (WFMH) declared a call for action on the mental health consequences of the manifold emergency in Syria. One of WFMH's concerns is the neglect of trauma created by the violence and disruption.

Multivariable logistic regression analyses found strong associations with depressive and anxiety symptoms for: female gender; the 18–25 age group; careers in private, business and ‘other’ sectors, as well as students; poor, moderate and good economic status; and having chronic disease(s). This last may be attributed to the higher incidence of depression and anxiety among chronically ill people than others.^11^ The COVID-19 pandemic is likely to add to the problem, given that people with chronic disease(s) are at increased risk of COVID-19 infection, which can be more severe, with increased rates of complications and mortality.^12^ The findings regarding young adults may be related to the fact that this age group, who were children when this war started, have grown up witnessing destruction and displacement and grave violations of children's rights – abductions, rape, killing and maiming continue unabated. Approximately 6 million Syrian children have been born since the crisis began, growing up knowing nothing but war, terror and displacement.^13^ Prolonged lockdowns that have harmed society, left many people unemployed, schools and universities closed and resulted in hyperinflation could explain the spread of mental disorders among younger generations. The findings related to females reflect the literature, which reports an increased prevalence of depression in women associated in part with female sex hormones and with factors such as life circumstances and culture.^14^ Also, this conflict has rendered Syrian women and girls particularly vulnerable to physical, sexual, economic and psychological violence.^2^

For the estimated high burden of depressive and anxiety symptoms encountered in this study, we must adopt the epidemiological modelling outputs for targeted mental health planning to calculate the required resources needed to guide optimal mental health service delivery.^15^

### Limitations

Our findings may not generalise to the general population, as well-educated Syrians of good socioeconomic status are over-represented in the sample. Elderly Syrians who were underrepresented in this study, which may be due to limited access to the internet are more likely to exhibit symptoms associated with depression and anxiety, as the elderly have a higher prevalence of chronic diseases.^11^ Self-reporting has certain limitations compared with structured interviews. The cross-sectional design of the study does not allow inferences to be drawn about causality.

## Data Availability

All data related to this paper's conclusions are available and stored by the authors. All data are available from the corresponding author on reasonable request.

## Supplementary material

For supplementary material accompanying this paper visit https://doi.org/10.1192/bji.2021.16. click here to view supplementary material
